# Personal

**Published:** 1917-09

**Authors:** 


### PERSONAL

---

Announcement of removal, travel, and other matters of interest are requested. Please report errors in the listing of any physician in the State and other directories, that we may co-operate with the State Society in securing a correct list.

---

Dr. Henry F. Senfter, formerly of the California State Sanitary Board, succeeds Dr. Edward Clark of Buffalo as State Sanitary Supervisor for the district, including Erie, Niagara, Orleans and Genesee Counties. His office will be in Ellicott Square, Buffalo. Dr. Clark goes to Albany as Acting Director of the Bureau of Child Hygiene.

---

Dr. Carlton R. Jewett of Buffalo has moved to 248 Linwood Ave.

---

Military Service: Dr. Floyd W. Haynes of Jamestown, 1st Lt., Medical, U. S. R., has been ordered from Ft. Benjamin Harrison, to service with the 3d N. Y. Artillery (formerly 65th Inf.), now 106 Reg. federal numbering. Drs. M. Emmett House and Theron B. Bond, of Cuba, were tendered a banquet, Aug. 13, having been ordered to training camp. Dr. C. H. Mackey of Lancaster was ordered to training camp, Aug 18. Dr. Victor A. Tyrasinski and Dr. Harry O. Maldinger of N. Tonawanda have been ordered to Ft. Benj. Harrison and to Washington, respectively.

---

Dr. Milton Bork of Buffalo has been appointed to the staff of the Anti-tuberculosis Society.

---

Dr. George Edward Fell of Buffalo is at present at 2469 Geneva Terrace, Chicago, and will be glad to hear from his friends, as he is still not fully recovered.

---

Dr. H. Neville has moved from Falconer to Jamestown.

